# Benefits and harms of breast cancer screening: Cohort study of breast cancer mortality and overdiagnosis

**DOI:** 10.1002/cam4.6373

**Published:** 2023-08-07

**Authors:** Sabrina Wang, Farhana Sultana, Anne Kavanagh, Carolyn Nickson, Amalia Karahalios, Lyle C. Gurrin, Dallas R. English

**Affiliations:** ^1^ Melbourne School of Population and Global Health University of Melbourne Melbourne Victoria Australia; ^2^ Cancer Epidemiology Division Cancer Council Victoria Melbourne Victoria Australia; ^3^ Telstra Health Melbourne Victoria Australia; ^4^ The Daffodil Centre The University of Sydney Sydney New South Wales Australia

**Keywords:** breast cancer, epidemiology and prevention, screening

## Abstract

**Background:**

Quantifying the benefits and harms of breast cancer screening accurately is important for planning and evaluating screening programs and for enabling women to make informed decisions about participation. However, few cohort studies have attempted to estimate benefit and harm simultaneously.

**Aims:**

We aimed to quantify the impact of mammographic screening on breast cancer mortality and overdiagnosis using a cohort of women invited to attend Australia's national screening program, BreastScreen.

**Methods:**

In a cohort of 41,330 women without prior breast cancer diagnosis, screening, or diagnostic procedures invited to attend BreastScreen Western Australia in 1994‐1995, we estimated the cumulative risk of breast cancer mortality and breast cancer incidence (invasive and ductal carcinoma in situ) from age 50 to 85 years for attenders and non‐attenders. Data were obtained by linking population‐based state and national health registries. Breast cancer mortality risks were estimated from a survival analysis that accounted for competing risk of death from other causes. Breast cancer risk for unscreened women was estimated by survival analysis, while accounting for competing causes of death. For screened women, breast cancer risk was the sum of risk of being diagnosed at first screen, estimated using logistic regression, and risk of diagnosis following a negative first screen estimated from a survival analysis.

**Results:**

For every 1,000 women 50 years old at first invitation to attend BreastScreen, there were 20 (95% CI 12‐30) fewer breast cancer deaths and 25 (95% CI 15‐35) more breast cancers diagnosed for women who attended than for non‐attendees by age 85. Of the breast cancers diagnosed in screened women, 21% (95% CI 13%‐27%) could be attributed to screening.

**Discussion:**

The estimated ratio of benefit to harm was consistent with, but slightly less favourable to screening than most other estimates from cohort studies.

**Conclusion:**

Women who participate in organised screening for breast cancer in Australia have substantially lower breast cancer mortality, while some screen‐detected cancers may be overdiagnosed.

## Graphical abstract

Using a cohort study, we estimated that for every 1000 women 50 years old at first invitation to attend BreastScreen Western Australia, by age 85 there were 20 (95% CI 12–30) fewer breast cancer deaths and 25 (95% CI 15–35) more breast cancers diagnosed for women who attended than for non‐attendees. Thus, for each breast cancer death avoided to age 85 by screening, 1.25 additional women had an over‐diagnosed cancer and were potentially treated unnecessarily..

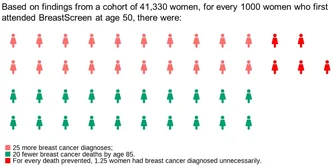

## 1 INTRODUCTION

Australia's organised mammographic screening program for breast cancer, BreastScreen, began in the 1990s following reports from randomised trials that screening reduced breast cancer mortality. BreastScreen originally screened women aged 50–69 every 2 years; women 70–74 were added to the target age range in 2013.

While randomised controlled trials are the most valid design to estimate the effect of introducing breast cancer screening, to quantify the benefits and harms of an established screening program such as BreastScreen, observational studies are necessary. The International Agency for Research on Cancer (IARC) convened a Working Group to produce a handbook on breast cancer screening.^1^, ^2^ As discussed in the handbook, cohort studies of women free of breast cancer at entry to the cohort are considered to give the most valid results.^1^

The IARC Working Group concluded there is sufficient evidence that mammographic screening of women age 50–74 reduces breast cancer mortality.^2^ A later systematic review of 38 European cohort studies also concluded organised screening reduces breast cancer mortality in all regions with established screening programs.^3^

Overdiagnosis—the phenomenon whereby screening detects cancer that would not have been diagnosed in a woman's lifetime—is a recognised harm of screening.^1^ Overdiagnosis leads to unnecessary treatment and the psychological distress of a cancer diagnosis. Quantifying the extent of overdiagnosis accurately is important because it influences women's decisions to participate in screening.^4^, ^5^, ^6^, ^7^ The preferred way to measure overdiagnosis is to compare the cumulative incidence (absolute risk) of breast cancer for women invited and not invited to screening (or women screened and unscreened).^8^ Calculating the risk instead of the rate to an age well beyond the end of screening overcomes the effect of lead time without making strong assumptions and avoids the compensatory drop in rates after screening ends.^8^ The risk is also not subject to selection bias inherent in the use of hazard ratios.^9^ However, most studies measuring the extent of overdiagnosis have been ecological studies, which estimate rates not risks and give widely varying estimates depending on the assumed length of time that screening advances the detection of cancer (i.e. the lead time).^10^ In contrast, cohort studies can be used to estimate risk, but of the 40 analyses of overdiagnosis described in the IARC handbook, only 10 were cohort studies.^1^ Several cohort studies and one case–control study were published after the IARC handbook^11^, ^12^, ^13^, ^14^, ^15^, ^16^; one by Lund et al.^13^ updated an earlier analysis.^17^

Quantifying the balance of benefits and harms of screening is important for helping women to make informed choices about whether to participate in screening, but estimates differ. A recent overview of systematic reviews stated that discrepancies in benefit‐to‐harm ratios will not be resolved without further original studies.^18^ Ideally, the ratio would be estimated within a single cohort study, since factors such as study design, population characteristics, underlying incidence rates and the design of screening programs could affect absolute estimates of breast cancer mortality and overdiagnosis. However, few cohort studies have assessed benefits and harms simultaneously.^11^, ^19^, ^20^

We aimed to quantify the impact of mammographic screening on breast cancer mortality and overdiagnosis using a cohort of women invited to attend BreastScreen Western Australia. To emulate the eligibility criteria for a randomised trial of screening, the analysis was restricted to women who had no previous screening or breast cancer diagnosis. We conducted an ‘as screened’ analysis by comparing breast cancer mortality and incidence between women who attended BreastScreen and women who did not. To provide estimates suitable for individual women considering screening,^21^ we estimated absolute risk differences in breast cancer mortality and diagnosis from age 50 (i.e. the age at which women are first invited to attend BreastScreen) to age 85 and the number of additional cancers diagnosed for each breast cancer death avoided.

## 2 METHODS

### 2.1 Participants

**FIGURE 1:**
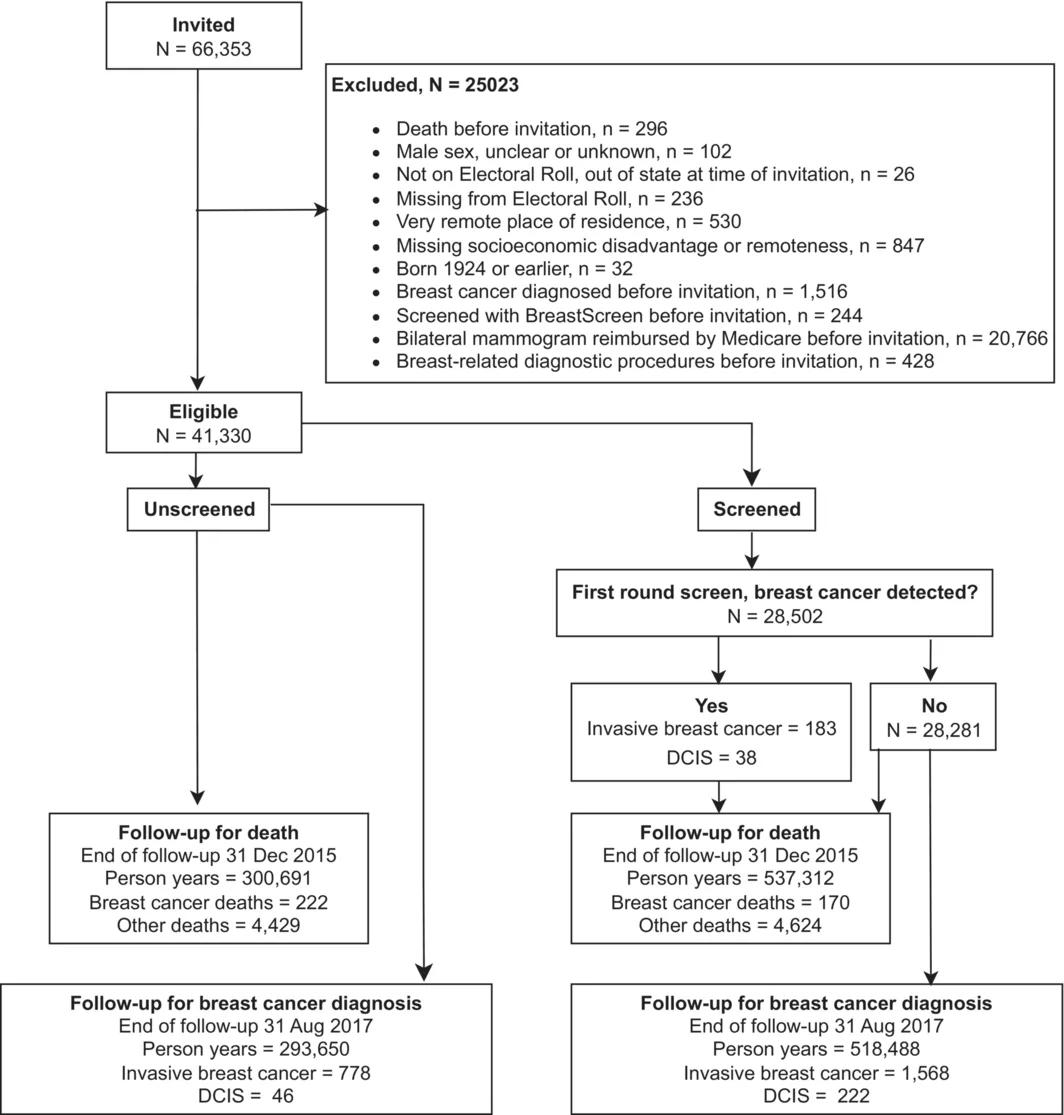
Flow diagram of participants.

The eligibility criteria were designed to emulate those of a randomised trial^22^ of de novo screening for which women who were previously screened or diagnosed with breast cancer would be ineligible. We began with a cohort of 66,353 women, aged 50–69, first invited by BreastScreen Western Australia to attend for screening between 11 May 1994 and 31 December 1995. BreastScreen obtained their names, dates of birth and addresses from the Western Australian Electoral Roll. We then excluded women diagnosed with breast cancer before invitation and women who, before their date of invitation, had screened with BreastScreen or had mammograms or other breast‐related diagnostic procedures reimbursed by Australia's national health insurance scheme agency, Medicare. We also excluded women missing covariate information or living in very remote areas because these areas have a high proportion of Indigenous women, and we had no data to control for Indigenous status. After exclusions, 41,330 women were included in the analyses (Figure 1).

### 2.2 Data

The Australian Institute of Health and Welfare (AIHW) linked various population and health‐related datasets and provided the data to the researchers. The Western Australia Department of Health datasets included data on each woman's status on the electoral roll, BreastScreen Western Australia screening history, diagnosis of breast cancer from 1982 as mandatorily reported to the cancer registry and death as recorded in the death registry. AIHW datasets included data on cancers and deaths in states and territories other than Western Australia from the Australian Cancer Database and the National Death Index. The AIHW also linked data on mammograms and other breast‐related diagnostic procedures reimbursed by Medicare. The AIHW used postcode of residence at the invitation to assign women an area‐based measure of socioeconomic disadvantage and a measure of remoteness of residence. For this, it used the Australian Bureau of Statistics' Index of Relative Socio‐economic Disadvantage^23^ in quintiles and the Accessibility and Remoteness Index of Australia classification^24^ for the place of residence (major cities (i.e. Perth), inner regional, outer regional, remote and very remote) derived from the 1996 census.

Year of birth was provided to the researchers in five‐year intervals (1925–29, 1930–34, …, 1945–49). We estimated the date of birth as the mid‐point of the interval (e.g. 30 June 1942 for women born 1940–44). For women born in 1945–49, the date of birth was set at 1 January 1945. Because the cohort was restricted to women 50–69 years of age at invitation, the cohort was already restricted to women born no later than early 1945.

### 2.3 Definition of screening

Women who were never screened were classified as unscreened throughout follow‐up. Women who attended BreastScreen were classified as unscreened from the date of invitation until the date of the first attendance for screening, and thereafter as screened, regardless of whether this was in response to the first invitation, a later invitation, or for any other reason. However, women who reported significant breast symptoms (pain, lump, nipple discharge) at their first attendance were classified as unscreened throughout follow‐up as were women who had been diagnosed with breast cancer after their date of invitation but before attending BreastScreen.

Some women had mammograms outside BreastScreen (i.e. reimbursed by Medicare) after their date of invitation but before date of diagnosis of any breast cancer. Because clinical indications for these mammograms were unavailable, we classified all Medicare‐reimbursed mammograms as non‐screening mammograms. To estimate the proportion that was potentially misclassified, we used cancer detection rates for BreastScreen and Medicare‐reimbursed mammograms and the positive predictive value of breast cancer symptoms^25^, ^26^ (Supplementary Materials 4).

### 2.4 Outcomes

Deaths from breast cancer were those where the underlying cause of death was breast cancer (International Classification of Diseases ICD‐10: C50; ICD‐9: 174). For analyses of breast cancer incidence, the primary outcome was invasive adenocarcinoma of the breast or ductal carcinoma in situ (DCIS). In a secondary analysis, DCIS was not included. DCIS was only recorded in the WA Cancer Registry.

### 2.5 Statistical analyses

We estimated the cumulative risk of death or diagnosis with breast cancer from age 50 to 85 years for screened and for unscreened women. From this, we derived the risk difference and risk ratio. For breast cancer incidence, we also estimated the attributable fraction, which is the proportion of breast cancers diagnosed in screened women that would not have been diagnosed without screening.

Adjusted risks were derived from statistical models that included year of birth, area‐based socioeconomic disadvantage and remoteness of residence. Estimates are presented for a reference group of women born in 1945 (i.e. age 50 at the time of invitation), residing in Perth and the middle quintile of socioeconomic disadvantage.

Survival analyses described below used flexible parametric survival analysis, which models the baseline cumulative hazard with cubic splines.^27^ To avoid bias when estimating risks,^28^ we accounted for competing causes of death.^29^ To facilitate calculating cumulative risk by age, attained age was the time scale.

We calculated 95% confidence intervals from 200 bootstrap replications of the analyses. All statistical analyses were performed using Stata 15.1 and 16.1 (StataCorp).

#### 2.5.1 Breast cancer mortality

Follow‐up for breast cancer mortality began at the date of invitation and ended at the earliest of the date of death or 31 December 2015 (the date for which cause of death coding was complete). To avoid immortal time bias, screening status was a time‐varying exposure and thus women who were screened had two records.^13^, ^30^ For the first record, which started at the date of invitation and ended at the date of first screen, they were classified as unscreened. For the second record, from the date of first screen until the end of follow‐up, they were classified as screened.

We calculated crude mortality rates and their ratio, and the predicted crude risks from age 50 to age 85 years, by multiplying the crude rates by 35 (the number of years from age 50 to 85). Adjusted risks of breast cancer death from age 50 to 85 years were estimated from the survival analysis.

#### 2.5.2 Breast cancer incidence (overdiagnosis)

Analysis of breast cancer incidence was more complicated than the analysis of mortality because some women had breast cancer detected at their first screen and hence contributed no person time to the screened group. To avoid bias when estimating the breast cancer risk for screened women, the risk was the sum of the risk of being diagnosed at the first screen and the risk of diagnosis following a negative first screen, calculated using the following equation, which is based on the approach used by Coldman and Phillips^31^ and where the risk is denoted by Prob (for probability):

(1) $$\begin{array}{c} \text{Prob}\left(\mathrm{BC}\right)=\text{Prob}\left(\mathrm{BC}\;\mathrm{at}\;\text{first screen}\right)\\ \;+\left(1-\text{Prob}\left(\mathrm{BC}\;\mathrm{at}\;\text{first screen}\right)\right)\\ \;\times \text{Prob}\left(\mathrm{BC}\;\text{after first screen}\right). \end{array}$$

We calculated crude cancer detection rates at first screen, and breast cancer incidence rates for unscreened women and for screened women who had a negative first screen. For screened women, we used Equation (1); the probability of cancer detected at first screen at age 50 was given by the cancer detection rate for women 50–54, and the crude risk after a first negative screen was 35 × incidence rate after the negative screen. For unscreened women, the crude predicted risk from age 50 to 85 was calculated as 35 × incidence rate. Before their first screen, screened women contributed person‐time to the unscreened group.

Adjusted risks were derived from logistic regression analysis and survival analysis. For screened women, the probability of detecting cancer at the first screen at age 50 was estimated using logistic regression, and the risk after a first negative screen was derived from a survival analysis. Women with a negative first screen were included in the survival analysis from the date of the first screen until the end of follow‐up, which was the earliest of date of diagnosis of breast cancer, date of death, or 31 August 2017 (when notification of breast cancer to the Western Australian Cancer Registry was complete). We then applied Equation (1) to estimate the adjusted risk of breast cancer diagnosis for screened women. To estimate the adjusted risk for unscreened women, screened women were included in the survival analysis as unscreened from the date of invitation until the first screen and never screened women were included from the date of invitation until the end of follow‐up.

#### 2.5.3 Quantitative bias analyses (sensitivity analyses)

As sensitivity analyses, we performed quantitative bias analyses to estimate the magnitude and direction of bias due to (1) misclassification of screening status and (2) confounding by two risk factors for breast cancer that might affect participation in BreastScreen, specifically family history of breast cancer and use of menopausal hormone therapy.^32^ External adjustment for confounding requires information on the prevalence of the risk factor in screened and unscreened women in the target population and the risk ratio for the confounder in relation to breast cancer.^33^ Family history and hormone therapy use were the only two risk factors for breast cancer for which we had information on prevalence in BreastScreen attendees and the general population. We used probabilistic analysis with 5000 Monte Carlo simulations.^33^ Random error and systematic error were combined to calculate 95% ‘total error’ intervals. Detailed methods are in Supplementary Materials 8.

## 3 RESULTS

### 3.1 Demographic characteristics of eligible women and BreastScreen attendance

The cohort included 41,330 eligible women invited to participate by BreastScreen between 11 May 1994 and 31 December 1995 (Figure 1). Almost half (45%) of the women were 50–55 years old at invitation and most (86%) lived in Perth. During follow‐up, 28,618 women attended BreastScreen, 116 were symptomatic at first attendance and were classified as unscreened, leaving 28,502 (69%) defined as screened.

Women who were younger, living in less socio‐economically disadvantaged areas and living in Perth were more likely to have been screened (Table 1). The median time from the date of invitation to the date of the first screen was 1.1 years (inter‐quartile range: 1.4 months–2.6 years). Most of the women who attended BreastScreen attended more than once (*n* = 24,745, 86.8%). The median number of screening rounds within the follow‐up period was five (inter‐quartile range: 3–8) and the maximum number was 20, corresponding to annual screening by some women. (During the period of the study, BreastScreen Western Australia performed annual screening for women with a strong family history of breast cancer.)

**TABLE 1:** Baseline characteristics of eligible women by screening status at the end of follow‐up.

	Screened (*n*, %)		Total
Total	28,502	69.0%	41,330
Year of birth (estimated baseline age, years)			
1945 (50)	6769	79.1%	8553
1940–1944 (51–55)	7571	75.9%	9979
1935–1939 (56–60)	6233	71.4%	8734
1930–1934 (61–65)	5031	62.9%	8004
1925–1929 (66–70)	2898	47.8%	6060
Index of Relative Socio‐economic Disadvantage (quintiles)			
Q1 (most disadvantaged)	5617	65.4%	8588
Q2	5870	67.9%	8640
Q3	5797	70.6%	8214
Q4	5617	70.6%	7951
Q5 (least disadvantaged)	5601	70.6%	7931
Accessibility and Remoteness Index			
Major cities (Perth)	24,639	69.7%	35,361
Inner regional	2069	67.5%	3065
Outer regional	1268	61.1%	2076
Remote	526	63.5%	828

### 3.2 Breast cancer mortality

For the analyses of breast cancer mortality, women were followed for a mean of 19.0 years to 31 December 2015. During 300,691 person‐years of unscreened time and 537,312 person‐years after screening, there were 222 and 170 deaths from breast cancer, respectively, (Figure 1; Table 2), including nine deaths in jurisdictions other than Western Australia. The crude rate ratio for breast cancer death, comparing screening with no screening, was 0.43 (CI 0.35–0.52) (Table 2). Women who attended BreastScreen also had a substantially lower rate of death from other causes (Table 2).

**TABLE 2:** Number of person years, deaths, breast cancer diagnoses and crude mortality and incidence rates by screening status.

Screening status	Deaths to 31 December 2015					Breast cancer diagnoses to 31 August 2017				
	Person years	Breast cancer		Other cause		Person years	Invasive	DCIS	Total	Rate^a^
		*N*	Rate^a^	*N*	Rate^a^					
Unscreened^b^	300,691	222	74	4429	1473	293,650	778	46	824	281
Screened	537,312	170	32	4624	861	NA^c^	1751	260	2011	
First screen		NA^c^		NA^c^		NA^c^	183	38	221	
After first screen	537,312	170	32	4624	861	518,488	1568	222	1790	345
Total	838,003	392	47	9053	1080	NA^c^	2529	306	2835	

Abbreviation: DCIS, ductal carcinoma in situ.

^a^ Crude rate per 100,000 person years.

^b^ Includes 61,878 person years accumulated by screened women before their first screen.

^c^ NA, not applicable. With respect to breast cancer diagnosis, women with cancer detected on the first screen contribute no person years of screened time. With respect to mortality, no deaths occur at the time of first screen.

The crude predicted risks from age 50 to age 85 were 11 per 1000 (1000 × 35 × 32/100,000) for women who screened at age 50 and 26 per 1000 for unscreened women (1000 × 35 × 74/100,000), giving a crude risk difference of 15 per 1000.

From the analysis that adjusted for potential confounders, for every 1000 women invited at age 50 and who started screening at that age, 14 deaths from breast cancer were predicted by age 85, compared with 34 deaths for women who did not screen (Table 3). The risk difference increased steadily with age, from 1 per 1000 (CI 1–2) at age 60 to 20 (CI 12–30) by age 85. The risk ratio was reasonably constant between ages 50 and 85 (0.42; CI 0.34–0.51 at age 85). (See Supplementary Materials 5 for details of the modelling).

**TABLE 3:** Cumulative risks, risk differences and risk ratios and attributable fractions for breast cancer mortality and breast cancer incidence from age 50 to specified ages. Estimates are shown for women aged 50 years at invitation and living in areas with the middle quintile of Index of Relative Socio‐economic Disadvantage and in Perth. Estimates are adjusted for of Index of Relative Socio‐economic Disadvantage, remoteness of residence and age.

Age (years)	Risk (per 1000)		Risk difference (per 1000)^a^ (95% CI)		Risk ratio^b^ (95% CI)		Attributable fraction (%) (95% CI)	
	Screened	Unscreened						
Breast cancer mortality								
60	1	2	1	(1–2)	0.41	(0.33–0.50)		
65	3	6	4	(2–5)	0.41	(0.33–0.51)		
70	4	11	6	(4–9)	0.41	(0.34–0.51)		
75	7	16	9	(6–14)	0.42	(0.34–0.51)		
80	9	22	13	(9–20)	0.42	(0.34–0.51)		
85	14	34	20	(12–30)	0.42	(0.34–0.51)		
Breast cancer incidence								
Invasive + ductal carcinoma only								
60	34	24	9	(7–12)	1.38	(1.25–1.54)	27.7	(20.0–34.9)
65	53	40	13	(9–17)	1.32	(1.20–1.46)	24.3	(16.6–31.7)
70	80	62	18	(11–23)	1.28	(1.18–1.42)	22.1	(14.9–29.5)
75	100	79	21	(13–27)	1.27	(1.17–1.40)	21.2	(14.2–28.4)
80	101	80	20	(14–31)	1.27	(1.16–1.39)	21.5	(13.4–27.9)
85	122	97	25	(15–35)	1.26	(1.15–1.37)	20.5	(13.0–26.9)
Invasive only								
60	28	22	6	(4–9)	1.28	(1.14–1.41)	21.6	(12.2–29.2)
65	45	37	8	(4–12)	1.22	(1.10–1.36)	18.0	(9.2–26.5)
70	69	58	11	(4–16)	1.19	(1.08–1.32)	15.7	(7.4–24.2)
75	87	74	13	(5–19)	1.17	(1.07–1.30)	14.8	(6.6–23.1)
80	89	75	12	(5–21)	1.18	(1.06–1.29)	15.0	(6.1–22.4)
85	107	92	15	(5–25)	1.16	(1.06–1.28)	14.1	(5.6–21.8)

Abbreviation: CI, confidence interval.

^a^ Risk in unscreened women ‐ risk in screened women.

^b^ Risk in screened women/risk in unscreened women.

### 3.3 Breast cancer incidence (overdiagnosis)

During a mean follow‐up of 19.7 years to 31 December 2017, 2835 women were diagnosed with breast cancer, including 2529 with invasive breast cancer (Figure 1; Table 2), of whom 62 were diagnosed outside Western Australia. Of the 2773 breast cancers diagnosed in Western Australia, 306 (11%) were DCIS, including 10 women who were subsequently diagnosed with invasive breast cancer (only the first diagnosis was included in the primary analysis). A higher proportion of cancers were DCIS in screened women compared with unscreened women (13% vs. 6%).

At their first BreastScreen visit, 183 women were diagnosed with invasive breast cancer and 38 with DCIS (Figure 1; Table 2). A further 1790 women were diagnosed with breast cancer at sometime during follow‐up after a negative first screen, with a crude rate of 345 per 100,000 years (Table 2). Another 824 breast cancers were diagnosed in unscreened women; the crude rate was 281 per 100,000 (Table 2).

The crude risk for unscreened women from age 50 to age 85 was 98 per 1000 (1000 × 35 × 281/100,000). The cancer detection rate at first screen for women 50–54 was 3.75 per 1000 (Supplementary Materials 6). Using Equation (1), the crude risk for women who began screening at age 50 was 124 per 1000 [1000 × ((3.75/1000) + (1–3.75/1000) × (35 × 345/100,000))]. The crude risk difference was 26 per 1000, the risk ratio was 1.26 and the attributable fraction was 20.6%.

Adjusted risks were derived from logistic regression and survival analysis. From the logistic regression, the estimated probability that a woman aged 50–54 had cancer detected at first screen was 3.7 (CI 2.6–5.1) per 1000 for invasive cancer plus DCIS. Applying Equation (1) to this probability and estimates from the survival analysis, we predicted that for every 1000 women who started screening at age 50, 122 (CI 101–146) would be diagnosed with breast cancer (invasive or DCIS) by age 85. From the survival analysis, we predicted that 97 (CI 78–119) unscreened women would be diagnosed with breast cancer (Table 3). The adjusted risk difference increased from 9 (CI 7–12) per 1000 at age 60 to 25 (CI 15–35) per 1000 by age 85. The adjusted risk ratio and the fraction of cases attributable to screening were highest at younger ages and declined gradually thereafter. By age 85, the adjusted risk of breast cancer was 1.26 (CI 1.15–1.37) times higher for screened women compared with unscreened women, and 20.5% (CI 13.0%–26.9%) of breast cancer diagnosed in screened women could be attributed to screening. (See Supplementary Materials 7 for modelling details).

When the outcome was restricted to invasive breast cancer only, the predicted probability of cancer detection at first screen at age 50 was 3.1 (CI 2.1–4.2) per 1000, and the association between screening and cancer diagnosis was weaker (Table 3). By age 85, the adjusted risk difference was 15 (CI 5–25) per 1000, the adjusted risk ratio was 1.16 (CI 1.06–1.28) and the adjusted fraction attributable to screening was 14.1% (CI 5.6%–21.8%).

### 3.4 Balance of benefit and harm (mortality reduction vs. overdiagnosis)

Based on the absolute decrease in risk of breast cancer death (20 per 1000) and excess risk of breast cancer diagnosis (25 per 1000) to age 85, we estimated that by age 85, for each death from breast cancer avoided due to screening, 1.25 additional women were diagnosed with invasive breast cancer or DCIS, 0.75 of whom were initially diagnosed with invasive breast cancer and 0.50 with DCIS.

### 3.5 Sensitivity analyses (quantitative bias analyses)

We estimated that of 3507 the 4496 women who had mammograms reimbursed by Medicare after their date of invitation (1935 before attending BreastScreen and 2561 who never attended BreastScreen), 3507 (78%) had diagnostic mammograms (Supplementary Materials 4). For our analysis, this would mean that 989 (2.4%) women were misclassified as unscreened for at least part of the follow‐up period. Thus, the estimated sensitivity of our classification of screening was 97.6%.

After adjusting for misclassification of screening status and confounding by family history, the bias‐adjusted risk ratio for mortality was 0.37 (total error interval: 0.30–0.45), the risk ratio for incidence was 1.19 (1.08–1.32) and attributable fraction was 16% (8%–24%) when comparing screened with unscreened women (Table 4; Supplementary Materials 8).

**TABLE 4:** Risk ratios and attributable fractions for risk of death and diagnosis of invasive breast cancer or ductal carcinoma in situ to age 85 adjusted for various biases as shown.

Bias adjusted for:	Mortality		Incidence			
	RR	(95% CI^a^)	RR	(95% CI)	AF (%)	(95% CI)
Observed (from Table 3)	0.42	(0.34–0.51)	1.26	(1.15–1.37)	20.5	(13.3–27.1)
Misclassification of screening status	0.40	(0.33–0.49)	1.29	(1.18–1.41)	22.5	(15.1–29.2)
Confounding by family history	0.39	(0.32–0.48)	1.16	(1.06–1.28)	14.1	(5.7–21.6)
Confounding by hormone therapy	0.39	(0.32–0.48)	1.15	(1.04–1.26)	12.8	(4.1–20.3)
Misclassification of screening status and confounding by family history	0.37	(0.30–0.45)	1.19	(1.08–1.32)	16.3	(7.6–24.0)
Misclassification of screening status and confounding by hormone therapy	0.37	(0.30–0.46)	1.17	(1.06–1.29)	14.7	(5.8–22.7)

Abbreviations: AF, attributable fraction; RR, risk ratio.

^a^ CI, confidence interval for the observed results; total error interval (random error plus systematic error) for all other rows.

After adjusting for misclassification of screening status and confounding by hormone therapy, the bias‐adjusted risk ratio for mortality was 0.37 (total error interval: 0.30–0.46), the risk ratio for incidence was 1.17 (1.06–1.29) and attributable fraction was 15% (6%–23%) when comparing screened with unscreened women (Table 4; Supplementary Materials 8).

## 4 DISCUSSION

For women aged 50 at their first invitation to attend BreastScreen, by age 85 the predicted risk of breast cancer death was 20 per 1000 lower for attendees than for non‐attendees while the predicted risk for breast cancer incidence was 25 per 1000 higher for attendees. For women who attended BreastScreen at age 50, about one in five breast cancers diagnosed by age 85 could be attributed to screening (i.e. were overdiagnosed). For each death avoided from breast cancer due to screening, 1.25 additional women were diagnosed with breast cancer (0.75 initially with invasive breast cancer and 0.50 with DCIS). After adjusting for misclassification of screening status and confounding by family history and hormone therapy, the estimated magnitude for mortality reduction was larger and the extent of overdiagnosis was smaller.

### 4.1 Strengths and limitations

The study used high‐quality data linkage, including national datasets to reduce selection bias due to interstate migration. While the Western Australian cancer registry was complete until August 2017, the Australian Cancer Database was complete only until July 2013 and does not include DCIS. Before that time, few cases were diagnosed outside Western Australia, thus few in total would have been missed. Linked Medicare data enabled us to exclude women who had mammograms before their first invitation, thereby emulating the eligibility criteria of a trial for which women who had previously been screened or diagnosed with breast cancer would be ineligible.^22^ Information on the indication for Medicare‐reimbursed mammograms was not available and we classified all Medicare‐reimbursed mammograms (i.e. outside BreastScreen) during follow‐up as non‐screening mammograms. Based on the substantially higher cancer detection rates following these mammograms and results from our quantitative bias analyses, the impact of misclassification in screening status was minimal.

Our analysis emulates an as‐screened analysis of a trial by comparing the risk of breast cancer death and diagnosis in attendees with non‐attendees in a cohort of women invited to BreastScreen. We handled the time‐varying nature of screening status by classifying the period before the first screen as unscreened, thus avoiding immortal time bias that would otherwise have underestimated the extent of overdiagnosis.^30^ Our method of estimating breast cancer risk for screened women appropriately dealt with cancers detected at first‐round screens, for which no person‐time is accrued after detection. Had women with cancers detected on their first screen been included in the survival analysis as screened, the estimate of overdiagnosis would have been inappropriately inflated. We accounted for competing risks of death, thereby avoiding bias in calculating risks from hazard ratios.^28^ A major advantage of presenting risks rather than rates for overdiagnosis is that they are not subject to the compensatory drop after screening ends.^8^

Although not all women in the analysis were followed until age 85, some were, so we did not have to extrapolate beyond the range of the data. We took care to choose models that fitted the relationship between age and breast cancer well (see Supplementary Materials).

We had no data on some potential confounders (i.e. known risk factors for breast cancer that might influence participation in screening) such as physical activity, adiposity, alcohol consumption and reproductive factors. We addressed confounding by family history and hormone therapy use through quantitative bias analyses. Our results indicate differential BreastScreen attendance according to these factors may have underestimated the magnitude of breast cancer mortality reduction and overestimated the extent of overdiagnosis.

Screened women had substantially lower mortality from causes other than breast cancer, consistent with previous cohort studies.^34^, ^35^ This suggests screened women had a lower prevalence of risk factors common to breast cancer and other causes of death (e.g. physical inactivity and adiposity); failure to account for these factors would have overestimated the extent of mortality reduction and underestimated overdiagnosis. Conversely, some other strong risk factors for death, including smoking, hypertension and elevated cholesterol, are not risk factors for breast cancer and not accounting for them thus does not bias our results.

### 4.2 Comparison with other studies

Comparison of studies on the impact of breast cancer screening programs is complicated by the heterogeneity in screening programs, study designs and analysis methods. We have focused our attention on cohort studies and case–control studies, since these give the most valid results of the observational designs.^1^ Cohort studies generally compare outcomes for invited versus non‐invited women, which is akin to an intention to treat analysis of a trial, or for attendees versus non‐attendees. Both methods require appropriate control for confounding to be valid. The latter is more relevant to women contemplating screening. For individual women, the most useful measure of association is the risk difference, and for overdiagnosis, additionally the attributable fraction. Risk differences, however, are more variable than risk ratios due to underlying differences in incidence and mortality. As discussed below, most studies report risk ratios or rate ratios.

### 4.3 Mortality

The most recent systematic review of studies on the impact of mammography screening on breast cancer mortality included 38 cohort studies from Europe. For 24 of these, women with breast cancer prior to study inception were excluded, as we did. We calculated the median rate ratio for breast cancer mortality in relation to the invitation to screening (yes/no) and to attendance (yes/no) to be 0.80 (interquartile range 0.74–0.86) and 0.58 (interquartile range 0.52–0.68), respectively.^3^

An Australian cohort study comparing screened and unscreened women reported a hazard ratio of 0.61 (CI  0.55–0.68) after adjusting for the same socioeconomic factors we included in our analysis; when restricted to previously unscreened women, the hazard ratio was 0.72 (CI 0.60–0.87).^36^ Two Australian case–control studies of breast cancer mortality reported odds ratios of 0.59 (CI 0.47–0.74)^37^ and 0.48 (CI 0.38–0.59),^38^ respectively, comparing women who attended BreastScreen with non‐participants. All three Australian studies would have included women who had mammograms reimbursed by Medicare‐ before BreastScreen began, which would attenuate the association with breast cancer mortality because women with a normal mammogram subsequently have lower incidence (and therefore mortality) of breast cancer.^39^ In summary, the reduction in risk we observed is stronger than in most other cohort studies of screening attendance and the two Australian case–control studies.

### 4.4 Overdiagnosis

Comparison of studies that estimate the extent of overdiagnosis is further challenged by different measures of overdiagnosis. Several studies report overdiagnosis as [risk ratio—1] or [rate ratio—1], which is known as the excess relative risk. Others, like us, report the attributable fraction, which we prefer because it estimates the probability that a cancer is overdiagnosed, and is readily interpretable by individual women. When the risk ratio is small, the two measures are similar (e.g. when the risk ratio is 1.10, the excess relative risk is 10% and the attributable fraction is 9%); when the risk ratio is large, the excess relative risk is larger than the attributable fraction (e.g. when the risk ratio is 1.5, the excess relative risk is 50% and the attributable fraction is 33%). As for mortality, the absolute risk difference provides a measure of the public health impact and is relevant for individual women.

The EUROSCREEN Working Group reviewed 13 European observational studies up to 2011 that it considered to be adequately adjusted for lead time and underlying incidence trend and reported the excess relative risk to be 6.5% (attributable fraction 6.1%).^40^ The IARC handbook included a further 17 studies between 2011 and 2014 and reported the excess relative risk to range from 2% to 22% (attributable fraction 2%–18%) for studies adequately adjusted for lead time and underlying incidence trend.^1^

Two more recent cohort studies of attendees and non‐attendees from Norway^13^ and Italy^12^ directly compared breast cancer incidence rates with relative risks of 1.04 and 1.10, respectively. The Norwegian study, which updated a previous analysis,^17^ was the only one that appeared to handle the time‐varying nature of screening status appropriately to avoid underestimating overdiagnosis due to immortal time bias. It was also notable for having controlled for several potential confounding variables. An earlier study, from Canada, appropriately dealt with immortal time and the first round (prevalent) screens issue, where women with cancer detected on the first screen contribute no screened person‐time.^31^ Naïve analyses in which cancers detected on the first screen are included in the numerator of the incidence rate for screened women would overestimate that rate and therefore overestimate overdiagnosis. In the Canadian study, the cumulative incidence from age 40 to 89 for screened and unscreened women was 163 and 139 per 1000, respectively, approximating to an attributable fraction of 14.7%. Another recent cohort study from Sweden of attendees and non‐attendees used a Markov model to estimate overdiagnosis and concluded it was minimal.^14^ A cohort study from Denmark of invitation reported almost no overdiagnosis.^11^, ^15^

For an Australian case–control study included in the IARC handbook,^41^ we calculated the odds ratio for all breast cancer in relation to ever having attended BreastScreen of 1.15 (CI 1.09–1.22), giving an attributable fraction of 13.4%. The study did not exclude women who previously had mammography or other breast‐related diagnostic procedures through Medicare.^41^ Women who have previously had a negative screen have low subsequent breast cancer risk^42^ and, as our results show, women who have been screened generally re‐attend for screening. This would attenuate the positive association between screening and risk of breast cancer diagnosis and thus underestimate the extent of overdiagnosis. The most recent study of overdiagnosis is a case–control study nested within the screening program in England. The odds ratio for ever having attended screening was 1.22 (CI 1.18–1.26) (attributable fraction 18%).^16^

Despite differences in comparison groups and methods, including biases, results from cohort studies and case–control studies are reasonably consistent, with attributable fractions around 20% or smaller.

### 4.5 Benefit versus harm: Breast cancer mortality versus overdiagnosis

We estimated that for each breast cancer death avoided by age 85 due to screening, 1.25 additional women had an over‐diagnosed cancer. This estimate is consistent with, but slightly less favourable to screening than most other published estimates. In 2012, the EUROSCREEN Working Group used data from various European sources to estimate that for 1000 women screened every 2 years from age 50–51 to 68–69, and followed until age 79, 7–9 breast cancer deaths were avoided and four additional women were diagnosed with breast cancer.^40^ These figures depended critically on several assumptions and the choice of estimates for mortality reduction and overdiagnosis used in the calculations. Puliti et al. used an Italian cohort of attendees and non‐attendees and reported that for each breast cancer death averted, no more than one additional case was diagnosed.^20^ Estimates from that study may be affected by immortal time bias, which would underestimate the extent of overdiagnosis and overestimate the mortality benefit; it also did not deal appropriately with the first round screen when estimating overdiagnosis. A Danish report combined results from four reports of cohort studies of mortality and overdiagnosis based on invitation to attend screening. It used methods based on the EUROSCREEN Working Group report and estimated that 2–3 breast cancer deaths were averted for each additional woman diagnosed with invasive breast cancer or DCIS^11^ Using averaged estimates from several Norwegian cohort studies of the effect of actual screening and of invitation, it was estimated that for each breast cancer death averted, there were an extra 1–2 women diagnosed with breast cancer.^19^

## 5 CONCLUSIONS

In a cohort of women without previous mammograms, we estimated the impact of screening on breast cancer mortality reduction and the extent of overdiagnosis by comparing attendees and non‐attendees. For each death avoided, we estimated that 1.25 additional women were diagnosed with breast cancer (0.75 initially with invasive breast cancer and 0.50 with DCIS).

## AUTHOR CONTRIBUTIONS

**Sabrina Wang:** Data curation (lead); formal analysis (lead); methodology (supporting); visualization (lead); writing – original draft (lead); writing – review and editing (equal). **Farhana Sultana:** Data curation (equal); formal analysis (supporting); methodology (supporting); writing – review and editing (supporting). **Anne Kavanagh:** Conceptualization (supporting); funding acquisition (supporting); methodology (supporting); writing – review and editing (supporting). **Carolyn Nickson:** Conceptualization (supporting); funding acquisition (supporting); methodology (supporting); writing – review and editing (supporting). **Amalia Karahalios:** Conceptualization (supporting); methodology (supporting); writing – review and editing (supporting). **Lyle C. Gurrin:** Conceptualization (supporting); methodology (supporting); writing – review and editing (supporting). **Dallas R. English:** Conceptualization (lead); formal analysis (supporting); funding acquisition (lead); investigation (lead); methodology (lead); project administration (lead); supervision (lead); writing – original draft (supporting); writing – review and editing (equal).

## FUNDING INFORMATION

Funding was provided by a NHMRC project grant (ID1063175) and the Australian Government Department of Health. The funders had no role in the study design, in the collection, analysis and interpretation of the data and the writing of the report. The NHMRC had no role in the decision to submit the paper for publication. The Department of Health had the right to disapprove publication if it would harm, prejudice or in any other way injure the interest it had in the intellectual property or if the manuscript contained its confidential information.

## CONFLICT OF INTEREST STATEMENT

During part of the time the study was conducted, Dallas English and Carolyn Nickson were members of the Technical Reference Group of BreastScreen Australia and Dallas English was a member of the Board of BreastScreen Victoria. Carolyn Nickson has received funding from the Australian Government to explore options for more risk‐based, personalised approaches to early detection of asymptomatic breast cancer in Australia. Sabrina Wang, Anne Kavanagh, Farhana Sultana, Lyle Gurrin and Amalia Karahalios have no interests to declare.

## ETHICS STATEMENT

The study protocol was approved by relevant Human Research Ethics Committees (see Supplementary Materials 3 for list) and data custodians and conforms with the Australian National Health and Medical Research Council's National Statement on Ethical Conduct in Human Research (2007). Because this was a retrospective cohort study, it was not practicable to obtain the individual's consent. Approval to conduct the research without seeking participant consent was obtained from the Human Research Ethics Committees acting in accordance with The Guidelines Under Section 95 of the Privacy Act 1988. The data were deposited, stored and analysed in the Secure Unified Research Environment facility. The researchers had no access to identifiers.

## Supporting information


Data S1.

## Data Availability

The data were provided to the researchers by the Australian Institute of Health and Welfare (AIHW). Researchers can apply to the AIHW, other data custodians and relevant Human Research Ethics Committees for permission to access the data. Further information is available on request from the corresponding author.
