# Supplementary material for: Benefits and harms of breast cancer screening: Cohort study of breast cancer mortality and overdiagnosis
Source: Cancer Med. 2023 Aug 7;12(17):18120–32. doi: 10.1002/cam4.6373 (PMC10524083; doi:10.1002/cam4.6373)
Supplement: Supplementary file 1 — Data S1. [file CAM4-12-18120-s001.docx]

**Benefits and harms of breast cancer screening:
cohort study of breast cancer mortality and overdiagnosis**

**Supplementary Material**

Table of contents

[1 List of supplementary tables 2](#_Toc78816541)

[2 List of supplementary figures 2](#_Toc78816542)

[3 Ethics 3](#_Toc78816543)

[4 Misclassification of screening status 3](#_Toc78816544)

[5 Details of models for mortality 6](#_Toc78816545)

[5.1 Breast Cancer mortality 6](#_Toc78816546)

[5.2 Death from other causes (competing risk) 7](#_Toc78816547)

[6 Breast cancers detected on first screen 9](#_Toc78816548)

[7 Details of models for breast cancer incidence 10](#_Toc78816549)

[7.1 Logistic regression model for cancer detection on first screen 10](#_Toc78816550)

[7.2 Flexible parametric survival model: Breast cancer incidence 10](#_Toc78816551)

[7.3 Flexible parametric survival model: death from other causes (competing risk) 11](#_Toc78816552)

[8 Quantitative bias analysis 14](#_Toc78816553)

[8.1 Misclassification of screening status 14](#_Toc78816554)

[8.2 Confounding 15](#_Toc78816555)

[8.3 Combined analysis of misclassification and confounding 17](#_Toc78816556)

[9 References 18](#_Toc78816557)

# List of supplementary tables

[Supplementary Table 1: BreastScreen and Medicare mammograms women who had Medicare mammograms during follow-up and number of breast cancer detected at first Medicare mammogram 3](#_Toc60836815)

[Supplementary Table 2: Breast cancers detected at first visit for BreastScreen and Medicare mammograms by age at first visit 5](#_Toc60836816)

[Supplementary Table 3: Goodness of fit of survival models for breast cancer mortality according to number of degrees of freedom for spline terms 6](#_Toc60836817)

[Supplementary Table 4: Goodness of fit for survival models of mortality from other causes, in analyses of breast cancer mortality 7](#_Toc60836818)

[Supplementary Table 5: Invasive breast cancer and ductal carcinoma in situ detected at first screen by age at first screen 9](#_Toc60836819)

[Supplementary Table 6: Goodness of fit for survival models of breast cancer incidence according to number of degrees of freedom for the spline terms 10](#_Toc60836820)

[Supplementary Table 7: Goodness of fit for survival models of mortality from other causes, in analyses of breast cancer incidence 11](#_Toc60836821)

[Supplementary Table 8: Synthetic two by two table replicating risk ratios from primary analyses of breast cancer mortality and incidence 14](#_Toc60836822)

[Supplementary Table 9: Synthetic two by two table for mortality assuming fixed misclassification of screening status 14](#_Toc60836823)

[Supplementary Table 10: Odds ratios and floating confidence intervals for family history of breast cancer (adapted from Beral et al. ^6^) 15](#_Toc60836824)

# List of supplementary figures

[Supplementary Figure 1: Hazard ratios for quintiles of IRSD in relation to breast cancer mortality 6](#_Toc60837090)

[Supplementary Figure 2: Cumulative hazard of breast cancer mortality according to complexity of model 7](#_Toc60837091)

[Supplementary Figure 3: Hazard ratios for year of birth in relation to breast cancer mortality 8](#_Toc60837092)

[Supplementary Figure 4: Cumulative hazards for breast cancer mortality for each covariate 8](#_Toc60837093)

[Supplementary Figure 5: Cumulative hazard of mortality from other causes according to complexity of model, in analyses of breast cancer incidence 11](#_Toc60837094)

[Supplementary Figure 6: Hazard ratios for year of birth in relation to mortality from other causes in analyses of breast cancer incidence 12](#_Toc60837095)

[Supplementary Figure 7: Cumulative hazard for death from other causes for each covariate in analyses of breast cancer incidence 12](#_Toc60837096)

# Ethics

The study protocol was approved by the following Human Research Ethics Committees: The University of Melbourne, Western Australian Department of Health, Commonwealth Department of Health, Australian Institute of Health and Welfare, Tasmania Health and Medical HREC, Australian Capital Territory Government Health, and NSW Health. The study was conducted without the consent of the participants but in accordance with the 10 Australian Privacy Principles. HRECs are required to assess such projects using guidelines under Section 95 of the Privacy Act 1988 (Cwth).

Approval for release of data was obtained from the following data custodians: BreastScreen WA, Health Department of WA, the NSW Cancer Institute, Tasmanian Cancer Registry, Victorian Cancer Registry, Queensland Government (Cancer Registry), South Australia Cancer Registry, Northern Territory Cancer Registry, ACT Cancer Registry, Australian Institute of Health and Welfare and the Commonwealth Department of Health (Medicare data).

# Misclassification of screening status

Women referred for a mammogram by their general practitioner or specialist can claim through Medicare, Australia’s national health insurance scheme. In this section, we estimate how many women were misclassified as unscreened because they had a mammogram for non-diagnostic purposes reimbursed by Medicare and quantify the direction and magnitude of bias in the estimated risk ratios (RRs). The misclassification applies to women whose only mammograms were reimbursed by Medicare and those whose first mammogram during follow-up was reimbursed by Medicare.

Unfortunately, it was not possible to classify individual mammograms as screening mammograms or otherwise because the clinical indication for the mammograms was not available. Throughout the period of this study, the text associated with the relevant Medicare item numbers stated that the item was applicable “if there is a reason to suspect the presence of malignancy because of: (i) the past occurrence of breast malignancy in the patient or members of the patient's family; or (ii) symptoms or indications of malignancy found on an examination of the patient by a medical practitioner.” Thus, it is possible that not all these mammograms would have been for investigations of signs or symptoms.

The maximum proportion of women who had potentially screened outside the BreastScreen program was estimated from the sum of women who claimed their first Medicare-reimbursed mammograms before their first BreastScreen mammographic screening and women who had mammograms through Medicare only.

Of the 41,330 eligible women, the maximum number who were misclassified as unscreened (for at least part of the follow-up period) was 4,496 (10.9%), assuming all women who either had their first Medicare mammogram before attending BreastScreen or had Medicare mammograms only were asymptomatic (Supplementary Table 1). Of these 4,496 women, 384 were diagnosed with breast cancer at their first Medicare mammogram.

Supplementary Table 1: BreastScreen and Medicare mammograms received by women who had Medicare mammograms during follow-up and number of breast cancer detected at first Medicare mammogram

| **Timing of mammogram relative to BreastScreen participation** | **Medicare bilateral mammograms after invitation** | **Breast cancer detected at first Medicare mammogram** |
| --- | --- | --- |
| Prior to first BreastScreen screen | 1,935 (20.5%) | 0* |
| Never attended BreastScreen | 2,561 (27.2%) | 384 |
| Following first BreastScreen screen | 4,923 (52.3%) | 422 |
| TOTAL | 9,419 (100%) | 806 |

* For attendance at BreastScreen to be considered as screening, no prior cancer must have been diagnosed.

To refine our estimate of the extent of misclassification, we compared the probability of having breast cancer detected on their first mammogram during the follow-up period for women whose first mammogram was performed by BreastScreen WA and women whose first mammogram was reimbursed by Medicare. The comparison assumes that a breast cancer is more likely to be diagnosed following a diagnostic mammogram than a screening mammogram. We used the following equation to estimate how many of the Medicare-reimbursed mammograms were done for diagnostic purposes:

|  | Pr(Cancer detected) = Pr(Cancer detected\|S)P(S) + Pr(Cancer detected\|D)Pr(D) | Eq 1 |
| --- | --- | --- |

Where

S = screening Medicare mammogram and

D = diagnostic Medicare mammogram.

Expressed in terms of numbers of cancers detected at first visit:

|  | Total cancer detected = Pr(Cancer detected\|S)N_S_ + Pr(Cancer detected\|D)N_D_ | Eq 2 |
| --- | --- | --- |

Where

N_S_ = number of screening Medicare mammograms and

N_D_ = number of diagnostic Medicare mammograms.

Given that the total number of Medicare mammograms (N_TOTAL_) is the sum of screening Medicare mammograms and diagnostic Medicare mammograms, the number of diagnostic Medicare mammograms can be expressed as:

|  | $N_{D}=\frac{Total cancer detected-\Pr\left( Cancer detected \vert S \right)N_{\mathrm{TOTAL}}}{\Pr\left( Cancer detected \vert D \right)-\Pr\left( Cancer detected \vert S \right)}$ | Eq 3 |
| --- | --- | --- |

We assumed that the probability of having cancer detected following a screening Medicare-reimbursed mammogram (i.e., Pr(Cancer detected|S)) was the same as the probability for a BreastScreen mammogram. This might be too low if most asymptomatic women had a family history of breast cancer.

We estimated the probability of having cancer detected at a diagnostic Medicare-reimbursed mammogram (i.e., Pr(Cancer detected|D)) from the positive predictive value of having breast cancer-related symptoms in combination with a diagnostic mammogram. In a report on mammograms performed on symptomatic women, the positive predictive values for any self-reported symptoms were 4.5% and 6.1% for self-reported lump.^1^ In a systematic review of positive predictive values for symptoms of patients attending primary care, the predictive values for breast cancer symptoms were 8.1%, 10.7% and 24.6% in three studies reviewed.^2^

We also used figures from the BreastScreen WA 1998 Statistical Report^3^ as a cross-check for the probability of having breast cancer detected at the first screening round.

Supplementary Table 2 shows the number of breast cancer detected per 10,000 first visits for women who had a BreastScreen mammogram first and women who had a Medicare-reimbursed mammogram first, compared with the 1998 statistical report from BreastScreen.^3^ The probability of having breast cancer detected at the first visit for women who had a BreastScreen mammogram first during follow-up was comparable to that in the BreastScreen report.^3^ In contrast, the probability of having breast cancer detected when the first mammogram was reimbursed by Medicare was about 10 times higher than for women whose first mammogram was performed by BreastScreen.

Supplementary Table 2: Breast cancers detected at first visit for BreastScreen and Medicare mammograms by age at first visit

| **Age at first visit (years)** | **Breast cancers detected at first visit (per 10,000 first visits)** | | |
| --- | --- | --- | --- |
|  | **BreastScreen WA 1998 Statistical Report^3^** | **BreastScreen WA first (n=26,567)** | **Medicare first**  **(n=4,496)** |
| **50-59** | 39 | 49 | 332 |
| **60-69** | 103 | 113 | 1161 |
| **70-79** | 134 | 215 | 1996 |
| **50-79** | 54 | 74 | 793 |

We then substituted these values into Equation 3 and used estimates of the positive predictive values of the combination of symptoms and diagnostic mammograms described above. If the positive predictive value were 10%, 3496 (78%) of the Medicare-reimbursed mammograms would be diagnostic. Based on this estimate, of the 4,496 (10.9%) of the cohort with possible screening mammograms outside the program, 989 (2.4%) of the eligible women should be classified as having had screening mammograms outside the BreastScreen program, meaning that we incorrectly classified them as unscreened in the analysis. We assumed the specificity of the measurement of screening was 100%.

# Details of models for mortality

## Breast Cancer mortality

### Regression model fit

Supplementary Table 3 shows the goodness of fit of each model and the likelihood ratio test results comparing models with different number of splines fitted for the baseline cumulative hazards. The model with three degrees of freedom for the splines had the lowest AIC whereas the model with one degree of freedom had the lowest BIC. There was evidence that the two model fits differed from each other (p=0.001). Thus, we chose the more complex model, which was the model with three degrees of freedom.

Supplementary Table 3: Goodness of fit of survival models for breast cancer mortality according to number of degrees of freedom for spline terms

| **Degrees of freedom for splines** | **Log likelihood** | **Total degrees of freedom** | **AIC** | **BIC** | **LRT^ (p-value)** |
| --- | --- | --- | --- | --- | --- |
| 1 | -1609 | 9 | 3236.21 | 3318.59 | - |
| 2 | -1606 | 10 | 3232.09 | 3323.63 | 0.013 |
| 3 | -1602 | 11 | 3225.53 | 3326.23 | 0.003 |
| 4 | -1601 | 12 | 3226.24 | 3336.09 | 0.256 |
| 5 | -1601 | 13 | 3227.15 | 3346.15 | 0.296 |

^Likelihood ratio test compared to the model from row above.

### Linearity assumption

The linearity assumption was assessed by a likelihood ratio test comparing models with each of the covariates IRSD, ARIA and year of birth fitted as categorical variables versus fitted as pseudo-continuous variables. There was little evidence of departure from linearity for ARIA (p=0.44) and year of birth (p=0.60). There was evidence of departure from linearity for IRSD (p=0.001), and the linearity assumption was further assessed visually by plotting the hazard ratios for each IRSD quintiles compared to most socioeconomically disadvantaged (quintile 1; Supplementary Figure 1).

Supplementary Figure 1: Hazard ratios for quintiles of IRSD in relation to breast cancer mortality

### Proportional hazards assumption

The proportional hazard assumptions were assessed by likelihood ratio tests comparing models with each of the covariates fitted with and without time-varying effects.^4^ The baseline cumulative hazard was fitted with three degrees of freedom and the time-varying effect was fitted with one and two degrees of freedom. When each covariate was fitted with a time-varying effect using one degree of freedom, there was little evidence that the proportional hazard was violated for screening (p=0.11), SEIFA (p=0.76), ARIA (p=0.88) or year of birth (p=0.28). When fitted using two degrees of freedom, there was also little evidence of violation for screening (p=0.27), SEIFA (p=0.40), ARIA (p=0.64) or year of birth (p=0.51).

## Death from other causes (competing risk)

### Regression model fit

Supplementary Table 4 shows the goodness of fit of each model fit and the likelihood ratio test results comparing models with different number of splines fitted for the baseline cumulative hazards for death from other causes. The model with four degrees of freedom had the lowest AIC whereas the model with two degrees of freedom had the lowest BIC. There was weak evidence that the two model fits differed from each other (p=0.021); plotting the baseline hazards shows the two models were very similar (Supplementary Figure 2). Thus, we opted for the simpler model with two degrees of freedom.

Supplementary Table 4: Goodness of fit for survival models of mortality from other causes, in analyses of breast cancer mortality

| **Degrees of freedom for splines** | **Log likelihood** | **Total degrees of freedom** | **AIC** | **BIC** | **LRT^ (p-value)** |
| --- | --- | --- | --- | --- | --- |
| 1 | -6338 | 9 | 12693.1 | 12775.48 | - |
| 2 | -6312 | 10 | 12644.68 | 12736.22 | <0.001 |
| 3 | -6311 | 11 | 12643.33 | 12744.02 | 0.067 |
| 4 | -6308 | 12 | 12640.86 | 12750.71 | 0.035 |
| 5 | -6308 | 13 | 12641.82 | 12760.82 | 0.308 |

^Likelihood ratio test compared to the model from row above.

Supplementary Figure 2: Cumulative hazard of mortality from other causes according to complexity of model


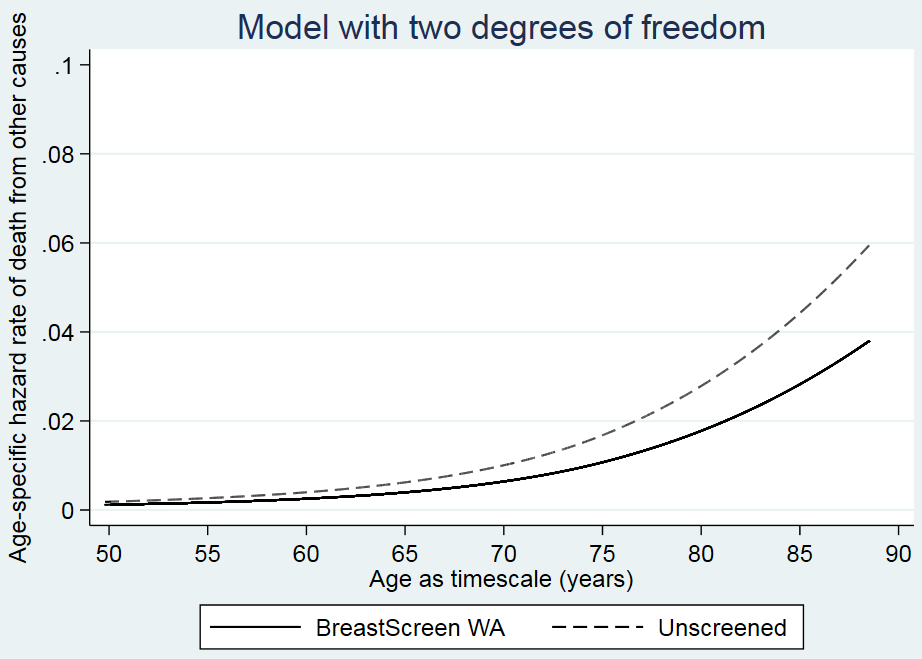

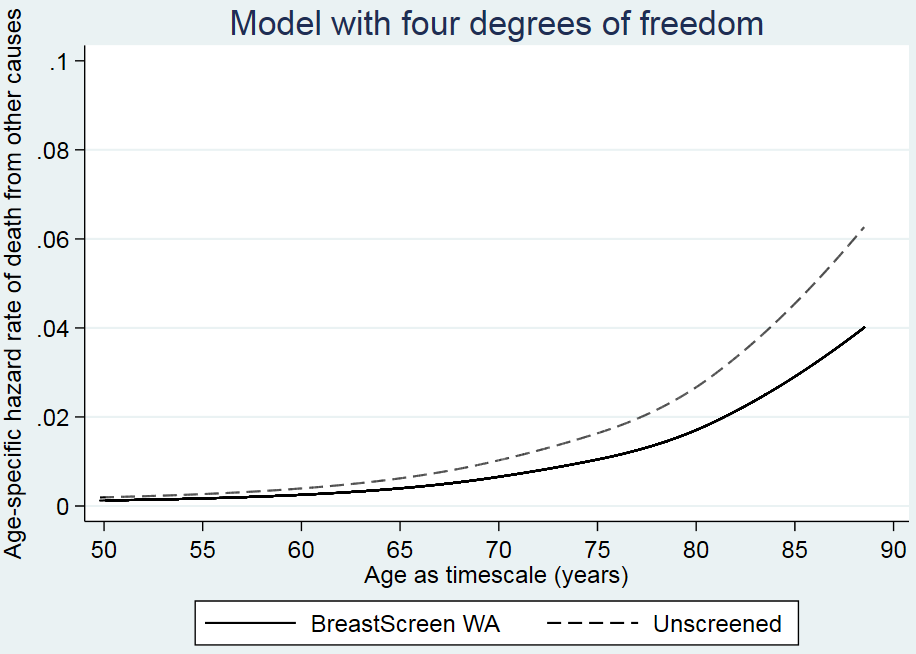


### Linearity assumption

The linearity assumption was assessed by a likelihood ratio test comparing models with each of the covariates IRSD, ARIA and year of birth fitted as categorical variables versus fitted as pseudo-continuous variables was performed. There was little evidence of departure from linearity for ARIA (p=0.32) and IRSD (p=0.046). Although there was evidence of departure from linearity for year of birth (p<0.001), the linearity assumption was further assessed visually by plotting the hazard ratios for each year of birth categories compared to women born in 1945 (Supplementary Figure 3). Year of birth did not appear to violate the linearity assumption.

Supplementary Figure 3: Hazard ratios for year of birth in relation to breast cancer mortality

### Proportional hazards assumption

The proportional hazard assumption was assessed by likelihood ratio tests comparing models with each of the covariates fitted with and without time-varying effects.^4^ The baseline cumulative hazard was fitted with three degrees of freedom and the time-varying covariate was fitted with one degree of freedom.

The likelihood-ratio tests comparing models with each of the covariates fitted with versus without time-varying effect suggest there was evidence that the proportional hazards assumption was violated for screening (p<0.001), IRSD (p<0.001), ARIA (p=0.001) or year of birth (p<0.001). However, due to the size of the sample, even a small deviation would result in small p-values. The proportional hazard assumption was further assessed visually by plotting the cumulative hazard of each covariate against age on a log scale (Supplementary Figure 4). The approximately parallel curves suggest there was limited evidence for violation of the assumption. The shape of the curves for year of birth is an artefact of using age as the analysis time scale.

Supplementary Figure 4: Cumulative hazards for breast cancer mortality for each covariate

| **Screening**  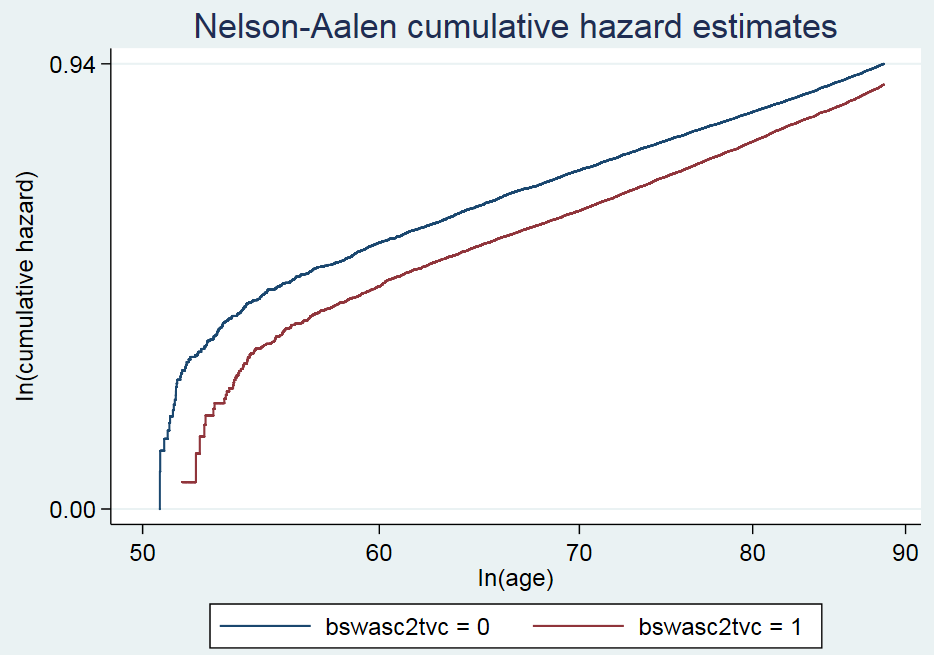 | **IRSD**  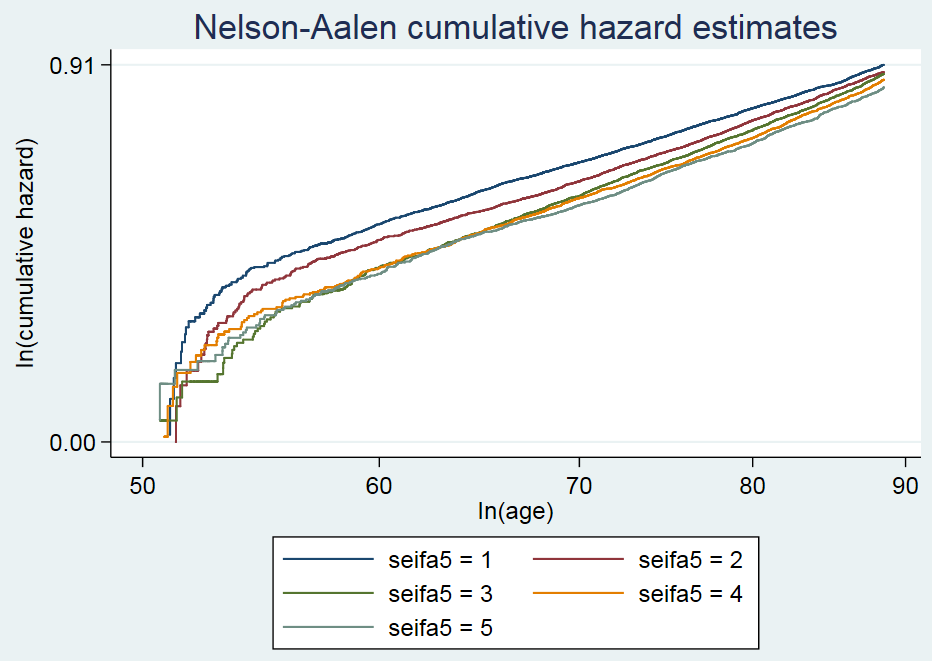 |
| --- | --- |
| **ARIA**  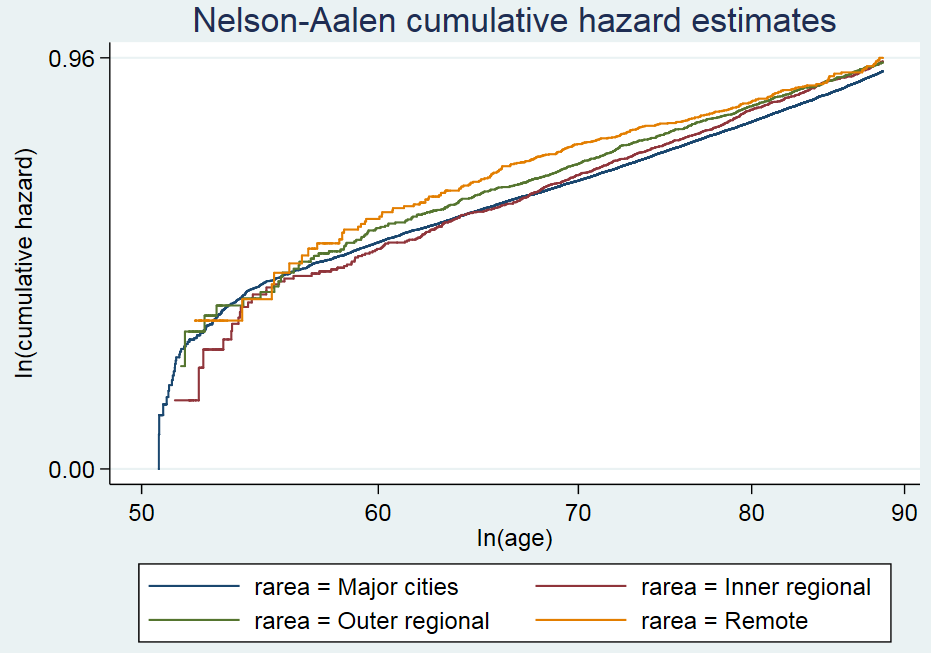 | **Year of birth**  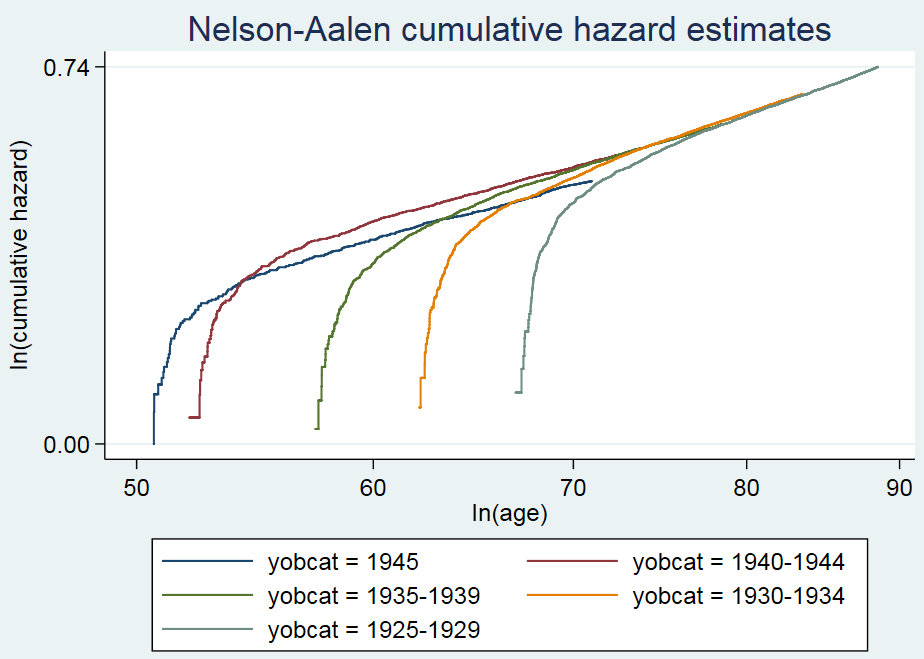 |

# Breast cancers detected on first screen

Supplementary Table 5: Invasive breast cancer and ductal carcinoma in situ detected at first screen by age at first screen

| Age 1^st^ screened | Breast cancer detected  Breast Cancer Diagnosis | | Number screened | Breast cancer detected  per 10,000 first screens | | |
| --- | --- | --- | --- | --- | --- | --- |
|  | Invasive | DCIS |  | Invasive | DCIS | Total |
| 50-54 | 33 | 6 | 10,394 | 31.7 | 5.8 | 37.5 |
| 55-59 | 40 | 11 | 7,382 | 54.2 | 14.9 | 69.1 |
| 60-64 | 49 | 10 | 6,064 | 80.8 | 16.5 | 97.3 |
| 65-69 | 46 | 11 | 3,953 | 116.4 | 27.8 | 144.2 |
| 70+^1^ | 15 | 0 | 709 | 211.6 | 0 | 211.6 |
| Total | 183 | 38 | 28502 | 64.2 | 13.3 | 77.5 |

DCIS=ductal carcinoma in situ

^1^117 women were first screened at age 75 or older

# Details of models for breast cancer incidence

## Logistic regression model for cancer detection on first screen

### Linearity assumption

The linearity assumption was assessed by likelihood ratio tests comparing models with each of the covariates IRSD, ARIA and age first screened fitted as a categorical variable versus a pseudo-continuous variable. There was no evidence of departure from linearity for IRSD (p=0.81), ARIA (p=0.21) or age first screened (p=0.88).

## Flexible parametric survival model: Breast cancer incidence

### Regression model fit

The table below shows the goodness of fit of each model fit and the likelihood ratio test results comparing models with different number of splines fitted for the baseline cumulative hazard for breast cancer incidence. The model with four degrees of freedom had the lowest AIC, whereas the model with three degrees of freedom had the lowest BIC. There was little evidence that the two models differed from each other (p=0.14). Thus, we opted for the simpler model, which was the model with three degrees of freedom.

Supplementary Table 6: Goodness of fit for survival models of breast cancer incidence according to number of degrees of freedom for the spline terms

| **Degrees of freedom for splines** | **Log likelihood** | **Total degrees of freedom** | **AIC** | **BIC** | **LRT^ (p-value)** |
| --- | --- | --- | --- | --- | --- |
| 1 | -6538 | 9 | 13093.26 | 13175.61 | - |
| 2 | -6524 | 10 | 13068.16 | 13159.67 | <0.001 |
| 3 | -6518 | 11 | 13058.00 | 13158.66 | 0.001 |
| 4 | -6518 | 12 | 13057.84 | 13167.65 | 0.142 |
| 5 | -6517 | 13 | 13060.45 | 13179.41 | 1 |

^Likelihood ratio test compared to the model from row above.

### Linearity assumption

The linearity assumption was assessed by likelihood ratio tests comparing models with each of the covariates IRSD, ARIA and year of birth fitted as a categorical variable versus a pseudo-continuous variable. There was no evidence of departure from linearity for IRSD (p=0.12), ARIA (p=0.78) or year of birth (p=0.13).

### Proportional hazard assumption

The proportional hazard assumption was assessed by likelihood ratio tests comparing models with each of the covariates fitted with and without time-varying effects. The baseline cumulative hazard was fitted with three degrees of freedom and the time-varying effect was fitted with one and two degrees of freedom. When each covariate was fitted with a time-varying effect using one degree of freedom, there was little evidence that the proportional hazard assumption was violated for screening (p=0.86), IRSD (p=0.69), ARIA (p=0.70) or year of birth (p=0.80). When fitted using two degrees of freedom, there was also no evidence of violation for screening (p=0.95), IRSD (p=0.78), ARIA (p=0.93) or year of birth (p=0.65).

## Flexible parametric survival model: death from other causes (competing risk)

### Regression model fit

The table below shows the goodness of fit of each model and the likelihood ratio test results comparing models with different number of splines fitted for the baseline cumulative hazards for death from causes other than breast cancer. The model with four degrees of freedom had the lowest AIC whereas the model with two degrees of freedom had the lowest BIC. There was weak evidence that the two model fits differed from each other (p=0.018); plotting the baseline hazards shows the two models were very similar (Supplementary Figure 5). Thus, we opted for the simpler model with two degrees of freedom.

Supplementary Table 7: Goodness of fit for survival models of mortality from other causes, in analyses of breast cancer incidence

| **Degrees of freedom for splines** | **Log likelihood** | **Total degrees of freedom** | **AIC** | **BIC** | **LRT^ (p-value)** |
| --- | --- | --- | --- | --- | --- |
| 1 | -6112 | 9 | 12241.09 | 12323.45 | - |
| 2 | -6080 | 10 | 12180.58 | 12272.09 | <0.001 |
| 3 | -6078 | 11 | 12177.32 | 12277.98 | 0.0218 |
| 4 | -6076 | 12 | 12176.56 | 12286.37 | 0.0963 |
| 5 | -6075 | 13 | 12176.92 | 12295.88 | 0.2014 |

^Likelihood ratio test compared to the model from row above.

Supplementary Figure 5: Cumulative hazard of mortality from other causes according to complexity of model, in analyses of breast cancer incidence


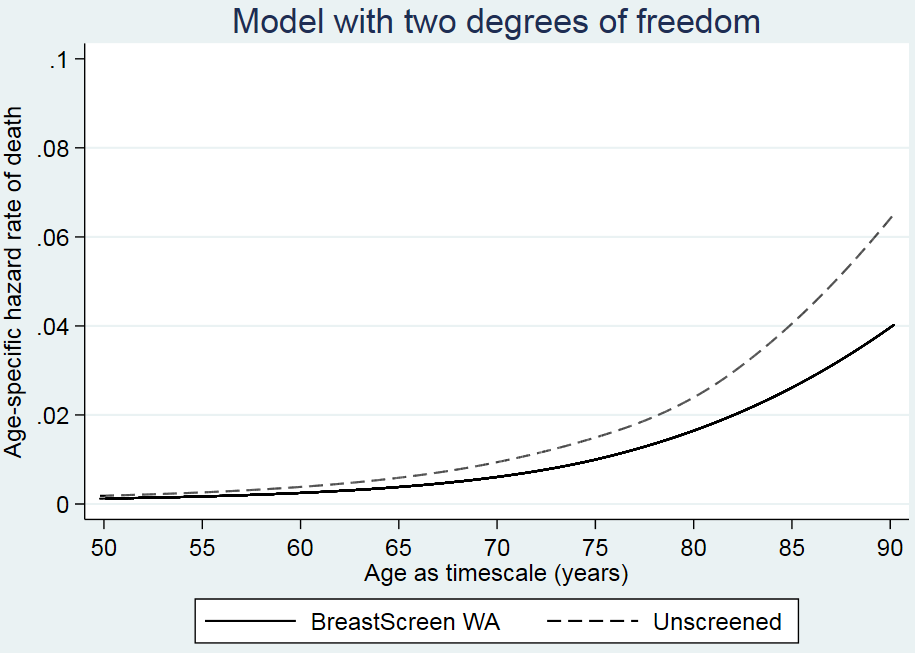

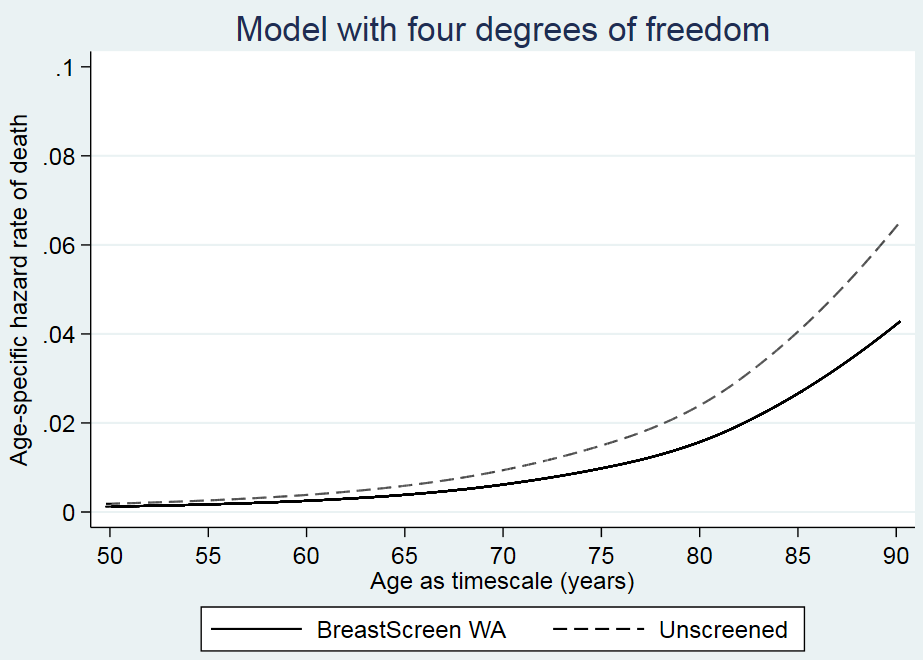


### Linearity assumption

The linearity assumption was assessed by likelihood ratio tests comparing models with each of the covariates SEIFA, ARIA and year of birth fitted as a categorical variable versus a pseudo-continuous variable. There was no evidence of departure from linearity for SEIFA (p=0.070) or ARIA (p=0.36). There was evidence of departure from linearity for year of birth (p<0.001). However, upon visual assessment, the association between year of birth and death appeared reasonably linear (Supplementary Figure 6).

Supplementary Figure 6: Hazard ratios for year of birth in relation to mortality from other causes in analyses of breast cancer incidence

### Proportional hazard assumption

The proportional hazard assumption was assessed by likelihood ratio tests comparing models with each of the covariates fitted with and without time-varying effects. The baseline cumulative hazard was fitted with two degrees of freedom and the time-varying covariate was fitted with one degree of freedom. Results suggest there was evidence of proportional hazard violation for screening (p<0.001), SEIFA (p<0.001), ARIA (p=0.004) and year of birth (p<0.001). However, due to the size of the dataset, even a small deviation would result in small p-values. The proportional hazard assumption was further assessed visually by plotting the cumulative hazard of each coverate against age on a log scale (Supplementary Figure 7). The approximately parallel curves suggest there is limited evidence for violation of the proprotional hazard assumption. The shape of the curves for year of birth is an artefact of using age as the analysis time scale.

Supplementary Figure 7: Cumulative hazard for death from other causes for each covariate in analyses of breast cancer incidence

| **Screening**  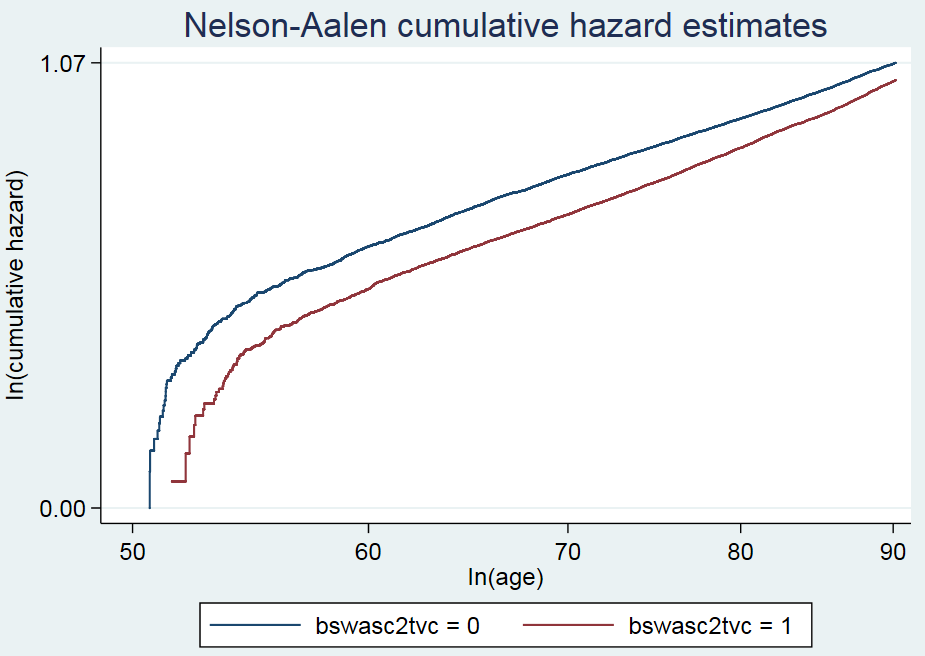 | **SEIFA**  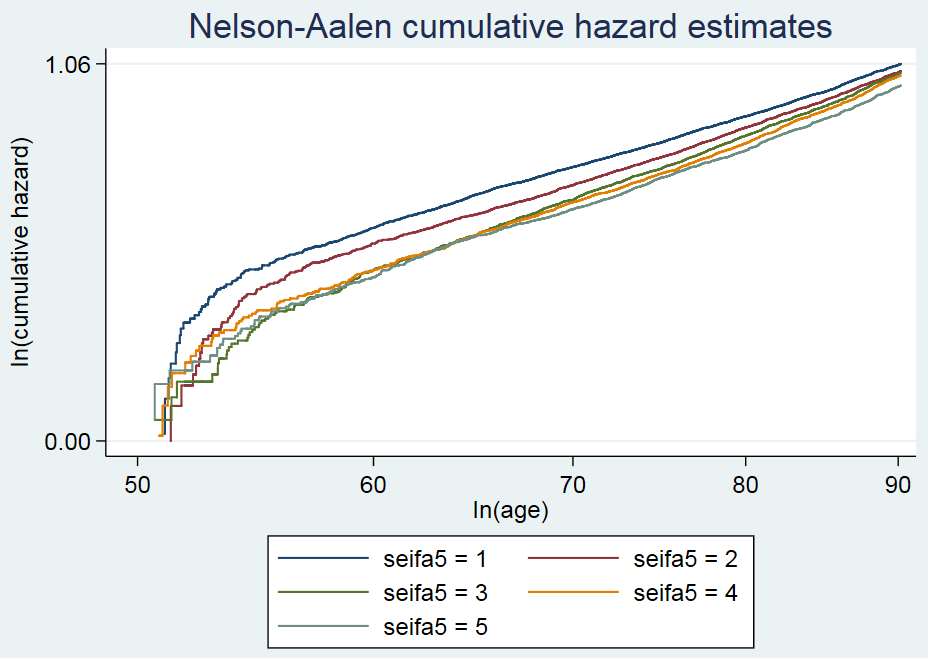 |
| --- | --- |
| **ARIA**  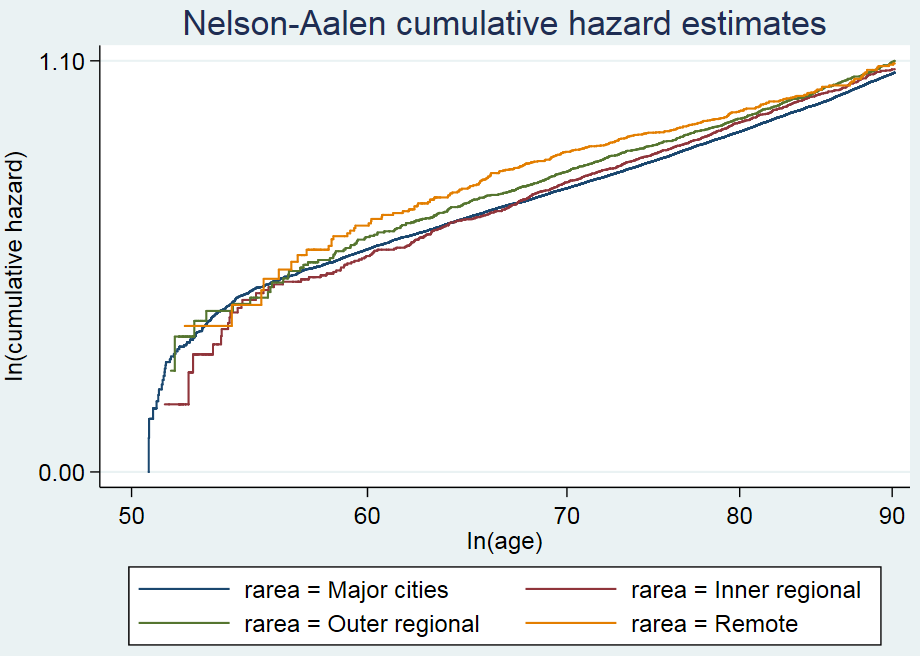 | **Year of birth**  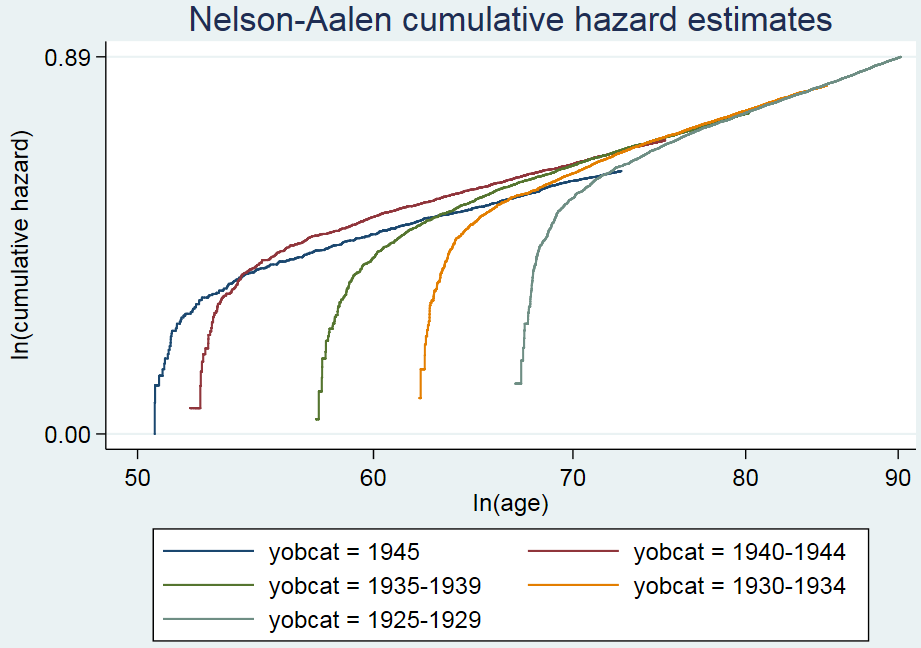 |

# Quantitative bias analysis

## Misclassification of screening status

To estimate the impact of misclassification we first had to construct synthetic 2 x 2 tables to approximate the results from the primary analyses. Supplementary Table 8 replicates the risk ratio (RR) and 95% confidence interval (CI) for breast cancer mortality and breast cancer incidence.

Supplementary Table 8: Synthetic two by two table replicating risk ratios from primary analyses of breast cancer mortality and incidence

|  | **Died from breast cancer** | | **Total** |  | **Diagnosed with breast cancer** | | | **Total** | |
| --- | --- | --- | --- | --- | --- | --- | --- | --- | --- |
|  | Yes | No |  |  | Yes | No |  | |  |
| Screened | 170 | 27000 | 27170 |  | 1742 | 24000 | 25742 | |  |
| Not screened | 222 | 14600 | 14822 |  | 667 | 11750 | 12417 | |  |
| Total | 392 | 41600 | 41992 |  | 2409 | 35750 | 38159 | |  |
|  |  |  |  |  |  |  |  | |  |
| Risk ratio | 0.42 (95% CI 0.34-0.51) | | |  | 1.26 (1.15-1.37) | | | |  |

The method then applies the sensitivity and specificity of the measurement of screening to calculate a “corrected” table. Assuming the sensitivity of measurement of screening is 97.6%, and the specificity is 100%, and applying formulae to estimate a “corrected” table^5^ gives Supplementary Table 9 for breast cancer mortality.

Supplementary Table 9: Synthetic two by two table for mortality assuming fixed misclassification of screening status

|  | **Died from breast cancer** | | **Total** |
| --- | --- | --- | --- |
|  | Yes | No |  |
| Screened | 174.2 | 27663.9 | 27838.1 |
| Not screened | 217.8 | 13936.1 | 14153.9 |
|  | 392 | 41600 | 41992 |

From this table, the bias-adjusted RR = 0.41. (As expected, the non-differential misclassification of the exposure resulted in (slightly) underestimating the effect of screening on mortality.)

In probabilistic analysis, the sensitivity is given a probability distribution. We assumed it had a uniform distribution, with minimum of 0.925 and maximum of 1.00. We then performed 5000 simulations in which the values of the sensitivity were chosen from this distribution.

For breast cancer incidence, we also estimated bias-adjusted attributable fractions and their total error intervals from the simulated risk ratios.

## Confounding

Because no information was available on risk factors for breast cancer other than the area-based measures of social disadvantage and access to services, quantitative bias analyses were performed to assess the possible impact on the results of unmeasured confounding. We used the following equation,^5^ which requires information on the relative risk for the unmeasured confounding variable and its prevalence in screened and unscreened women. The formula is:

$${RR}_{adj}= {RR}_{obs}\frac{{RR}_{CD}p_{0}+1-p_{0}}{{RR}_{CD}p_{1}+1-p_{1}}$$

Where

RR_adj_ = RR for the exposure related to the outcome after adjusting for the confounder, RR_obs_ is the observed RR without adjustment, RR_CD_ = RR between the confounder and the outcome, p_1_ = proportion of screened participants for which the confounder = 1 (e.g. family history) and p_0_ is the number of unscreened participants for which the confounder = 0 (e.g., no family history).

### Family history of breast cancer

Apart from age, a family history of breast cancer is among the strongest risk factors for the disease and likely to be a determinant of women’s screening behaviour. The Table below is adapted from a pooled analysis of 52 case-control studies of family history and risk of breast cancer.^6^ The confidence intervals presented in the paper were calculated using the floating absolute risk method.^7^ We calculated the odds ratio for any family history from these data. Because the odds ratios estimated using the floating absolute risk approach are approximately uncorrelated,^7^ the categories of 1, 2, and 3 or more affected relatives were combined using standard fixed effects meta-analysis. To calculate the conventional confidence interval for any family history, the floating variances for “None” and “Any” were added.^8^ The pooled odds ratio is thus 1.87 (95% CI 1.78-1.96). For the probabilistic bias analysis, we assumed that this risk ratio was log normally distributed with mean ln(1.87) = 0.627 and scale parameter (standard deviation) of 0.025.

Supplementary Table 10: Odds ratios and floating confidence intervals for family history of breast cancer (adapted from Beral et al. ^6^)

| **Number of first-degree relatives with breast cancer** | **Cases** | **Controls** | **Odds ratio** | **99% Floating confidence interval** |
| --- | --- | --- | --- | --- |
| None | 50713 | 94548 | 1.00 | 0.97-1.03 |
| 1 | 6810 | 6998 | 1.80 | 1.70-1.91 |
| 2 | 603 | 404 | 2.93 | 2.37-3.63 |
| 3 or more | 83 | 36 | 3.90 | 2.03-7.49 |
|  |  |  |  |  |
| Any family history* | 7496 | 7438 | 1.87 |  |

* Calculated by us

About 16% of women who attended BreastScreen WA in 1997-98 reported “some” family history of breast cancer.^3^ The prevalence of a family history for non-attendees is unknown and Australian data on the prevalence of a family history are sparse. 45 and Up is a large cohort study of about 250,000 people aged 45 years or older living in New South Wales.^9^ Participants were asked at baseline (2006-2009) about family history of breast cancer: 6.8% reported their mother had breast cancer and 5% reported a sister with breast cancer. The lowest estimate of the prevalence of a first-degree family history is therefore 6.8% and the highest estimate about 12%.

We also used data from control women in the Australian Breast Cancer Family Study to estimate the prevalence of a family history of breast cancer. This population-based case-control-family study of breast cancer was conducted in Melbourne and Sydney from 1996 to 1999 (Gillian Dite, personal communication, 31 January 2013). Control women were identified from Electoral Rolls. 67% of women aged 50-59 years participated. Twelve percent (95% CI 8% to 17%) of the women reported a first-degree relative with breast cancer. No data were available for older women.

Finally, questions relevant to breast cancer screening were included in the 2012 South Australia Health Omnibus, an annual face-to-face interview survey.^10^ Data were obtained from 1162 women aged 40-84 years. The participation fraction was 70%. The prevalence of a family history (first-degree relatives and aunts) was 32% for women who reported that they had ever attended BreastScreen SA and 30% for women who had never attended. Inclusion of aunts would give a higher estimate of women with a family history. Because these estimates were not compatible with other sources they were not used. For example, in the pooled analysis of 52 case-control studies of family history of breast cancer, 13% of cases and 7% of controls reported a first-degree family history.^6^

We assumed the prevalence of a first degree family history in BreastScreen attendees was 16% and the participation rate was 53%.^3^ We assumed the prevalence of a first-degree family history to be 12% in the total population. The prevalence in the total population is a weighted average of the prevalence for unscreened women and that for unscreened women, where the weight is the proportion screened:

P_t_ = S × P_s_ + (1-S) × P_u_

Rearranging this formula gives the prevalence in unscreened women as:

P_u_ = (P_t_ – (S × P_s_))/(1-S) = (0.12 - (0.53 × 0.16)/(1-0.53) = 0.07 or 7%.

For the probabilistic bias analysis, we assumed that the prevalence of a family history was fixed at 0.16 for screened women, and that it followed a uniform distribution with minimum value of 0.03 and maximum of 0.10 for unscreened women. We performed 5000 Monte Carlo simulations.

### Hormone therapy use

Use of menopausal hormone therapy increases the risk of breast cancer, particularly use of combined estrogen- progestogen therapy, which is prescribed to women who have an intact uterus. A pooled analysis of cohort studies of breast cancer incidence found risk ratios of 2.08 (95% CI 2.02–2.15) for combined therapy and 1.33, 1.28–1.37 for oestrogen-only in relation to current use of between 5 and 14 years.^11^ In the 1989-90 Australian National Health Survey, about 15% of women 50-54 years of age had had a hysterectomy,^12^ and so we assumed that 85% of women using hormone therapy would use combined therapy. We calculated a weighted average for all types of hormone therapy as 1.95 (95% CI 1.88-2.01). In 2008, the median duration of use by Australian women was 10 years.^13^ For the probabilistic bias analysis for breast cancer incidence, we assumed the risk ratio for hormone therapy and breast cancer was log-normally distributed with mean ln(1.95) = 0.67 and standard deviation of 0.017.

Risk ratios for breast cancer death are from the Million Women’s Study.^11^ For use of 5 or more years, the risk ratios were 1.64 (95% CI 1.52-1.76) for combined therapy and 1.35 (1.24-1.47) for estrogen only therapy. We combined these to give an overall RR of 1.59 (1.46-1.73). For the probabilistic bias analysis for mortality, we assumed the risk ratio for hormone therapy and breast cancer was log-normally distributed with mean ln(1.59) = 0.47 and standard deviation of 0.043.

In 1997/8, 36% of women age 50-69 attending BreastScreen WA reported using hormonal therapy in the three months before attendance.^3^ In the 1995 National Health Survey, 30% of women age 55-64 reported using hormone therapy in the previous three months.^14^ The National Health Survey had > 90% participation. We estimated the prevalence of current use of hormone therapy in unscreened women:

P_u_ = (P_t_ – (S × P_s_))/(1-S) = (0.30 - (0.53 × 0.36)/(1-0.53) = 0.23 or 23%.

For the probabilistic analysis, we used uniform distributions for the prevalence of hormone therapy use. For screened women, the distribution had a minimum value of 0.33 and maximum value of 0.39. For unscreened women, the lower limit was 0.20 and maximum value was 0.26.

## Combined analysis of misclassification and confounding

Finally, we estimated the combined impact of misclassification of screening status and confounding. First, the effect of misclassification was estimated, then the impact of confounding.^15^

# References

1 Barlow WE, Lehman CD, Zheng Y, Ballard-Barbash R, Yankaskas BC, Cutter GR, Carney PA, Geller BM, Rosenberg R, Kerlikowske K, Weaver DL, Taplin SH. Performance of diagnostic mammography for women with signs or symptoms of breast cancer. *J Natl Cancer Inst* 2002;94: 1151-9.

2 Shapley M, Mansell G, Jordan JL, Jordan KP. Positive predictive values of >/=5% in primary care for cancer: systematic review. *The British journal of general practice : the journal of the Royal College of General Practitioners* 2010;60: e366-77.

3 BreastScreen WA Public Health Division Health Department of Western Australia, BreastScreen WA 1997-1998 Statistical Report, 1998.

4 Royston P, Lambert PC. *Flexible Parametric Survival Analysis Using Stata: Beyond the Cox Model*ed. College Station: Stata Press, 2011.

5 Lash TL, Fox MP, Fink AK. *Applying Quantitative Bias Analysis to Epidemiologic Data*ed. Dordrecht: Springer, 2009.

6 Collaborative Group on Hormonal Factors in Breast Cancer. Familial breast cancer: collaborative reanalysis of individual data from 52 epidemiological studies including 58,209 women with breast cancer and 101,986 women without the disease. *Lancet* 2001;358: 1389-99.

7 Easton DF, Peto J, Babiker AG. Floating absolute risk: an alternative to relative risk in survival and case-control analysis avoiding an arbitrary reference group. *Statistics in medicine* 1991;10: 1025-35.

8 Orsini N. From floated to conventional confidence intervals for the relative risks based on published dose-response data. *Comput Methods Programs Biomed* 2010;98: 90-3.

9 Banks E, Redman S, Jorm L, Armstrong B, Bauman A, Beard J, Beral V, Byles J, Corbett S, Cumming R, Harris M, Sitas F, Smith W, Taylor L, Wutzke S, Lujic S. Cohort profile: the 45 and up study. *Int J Epidemiol* 2008;37: 941-7.

10 Beckmann KR, Roder DM, Hiller JE, Farshid G, Lynch JW. Do breast cancer risk factors differ among those who do and do not undertake mammography screening? *Journal of medical screening* 2013;20: 208-19.

11 Collaborative Group on Hormonal Factors in Breast Cancer. Type and timing of menopausal hormone therapy and breast cancer risk: individual participant meta-analysis of the worldwide epidemiological evidence. *Lancet* 2019;394: 1159-68.

12 Australian Bureau of Statistics, Women's Health. ABS Catalog Number 4365.0, 1994.

13 MacLennan AH, Gill TK, Broadbent JL, Taylor AW. Continuing decline in hormone therapy use: population trends over 17 years. *Climacteric : the journal of the International Menopause Society* 2009;12: 122-30.

14 Australian Bureau of Statistics, National Health Survey 1995: summary of results. ABS Catalog No. 4364.0. Australian Bureau of Statistics, 1997.

15 Orsini N, Bellocco R, Bottai M, Wolk A, Greenland S. A tool for deterministic and probabilistic sensitivity analysis of epidemiologic studies. *Stata J* 2008;8: 29-48.
